# Ultrasensitive and bifunctional ZnO nanoplates for an oxidative electrochemical and chemical sensor of NO_2_: implications towards environmental monitoring of the nitrite reaction†

**DOI:** 10.1039/c8ra01358f

**Published:** 2018-03-21

**Authors:** Shivsharan M. Mali, Parag P. Chavan, Yuvraj H. Navale, Vikas B. Patil, Bhaskar R. Sathe

**Affiliations:** Department of Chemistry, Dr Babasaheb Ambedkar Marathwada University Aurangabad 431004 Maharashtra India bhaskarsathe@gmail.com; Functional Materials Research Laboratory, School of Physical Sciences, Solapur University Solapur 413255 Maharashtra India

## Abstract

Herein, we focused on the one pot synthesis of ZnO nanoplates (NP edge thickness of ∼100 nm) using a chemical emulsion approach for chemical (direct) and electrochemical (indirect) determination of NO_2_. The structural and morphological elucidation of the as-synthesized ZnO NPs was carried out by X-ray diffraction (XRD), scanning electron microscopy (SEM), energy dispersive analysis of X-ray (EDAX), thermogravimetric analysis (TGA) and BET-surface area measurements. The XRD studies of the as-synthesised NPs reveal that ZnO NPs have a Wurtzite type crystal structure with a crystallite size of ∼100 nm. Such ZnO NPs were found to be highly sensitive to NO_2_ gas at an operating temperature of 200 °C. Electrocatalytic abilities of these ZnO NPs towards NO_2_/NO_2_^−^ were verified through cyclic voltammetry (CV) and linear sweep voltammetry (LSV) using aqueous 1 mM NO_2_^−^ (nitrite) in phosphate buffer (pH 7) solution. The results revealed enhanced activity at an onset potential of 0.60 V *vs.* RCE, achieved at a current density of 0.14 mA cm^−2^. These ZnO NPs show selective NO_2_ detection in the presence of other reactive species including CO, SO_2_, CH_3_OH and Cl_2_. These obtained results show that this chemical route is a low cost and promising method for ZnO NPs synthesis and recommend further exploration into its applicability towards tunable electrochemical as well as solid state gas sensing of other toxic gases.

## Abstract 2

Herein, we focused on the one pot synthesis of ZnO nanoplates (NP edge thickness of ∼100 nm) using a chemical emulsion approach for chemical (direct) and electrochemical (indirect) determination of NO_2_.

## Graphical abstract

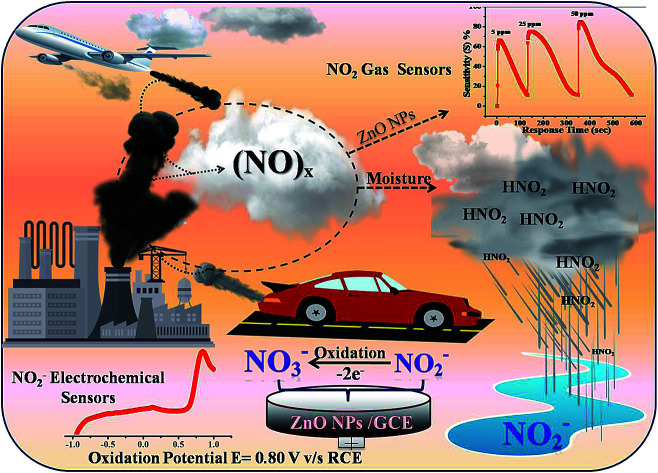

## 1 Introduction

Nitrogen dioxide (NO_2_) gas is well known to be one of the irritant gases, and is a prominent intermediate product of the industrial synthesis of nitric acid. Moreover, other common and considerable contributors to NO_2_ gas production are combustion engines, the burning of fossil fuels, fertiliser industries, cigarette smoke, and butane and kerosene heaters and stoves.^1^ Unfortunately, NO_2_ can cause respiratory infections, photochemical smog and acid rain^2^ and it is injurious to human health. Exposure to unsafe and elevated levels in the body can cause severe underlying diseases such as chronic obstructive pulmonary disease or asthma. For example, NO_2_ reacts with water droplets in the trachea and lungs forming droplets of nitric acid and these tiny droplets penetrate deep into the lungs causing various respiratory diseases.^3^ Moreover, NO_2_ exposure has also been associated with sudden infant death syndrome.^4^ Thus, it is imperative to develop a sensor for detecting NO_2_ gas. Significantly, many solid state gas sensors have been recently explored for NO_2_ gas sensing such as WO_3_,^5^ VO_2_,^6^ NiO,^7^ SnO_2_ (ref. 8) and ZnO.^9^ Among these, ZnO is a cheap, stable and nontoxic material and it is possible to further improve its chemical and physical properties by controlling its dimensions in a micro/nano-regime. This motivated us to develop a new, cost effective, safer synthetic method for the synthesis of its nanostructures by a chemical approach, taking into consideration energy and environmental factors. The past literature reflects that the properties and performances of ZnO based devices are significantly influenced by its structural features.^10,11^ Recent studies in the literature have demonstrated that the crystal structure and its morphology have a significant influence on its surface sensitive reactions, especially gas-sensing, electronic, electrochemical and many more.^12,13^ For example, one-dimensional (1D) nanostructures of ZnO, such as nanowires,^14^ nanorods,^15^ and nanobelts^16^ and their hierarchical structures were widely used in gas sensor applications,^15^ also, recently, two-dimensional (2D) structures, such as NPs, have been another common structure of ZnO.^17,18^ Thus, the need for simple and cost effective ZnO based gas sensors further encourages us to design and develop a method for the synthesis of ZnO NPs with a high surface area and explore it further for NO_2_ gas sensing studies.

**Scheme 1:**
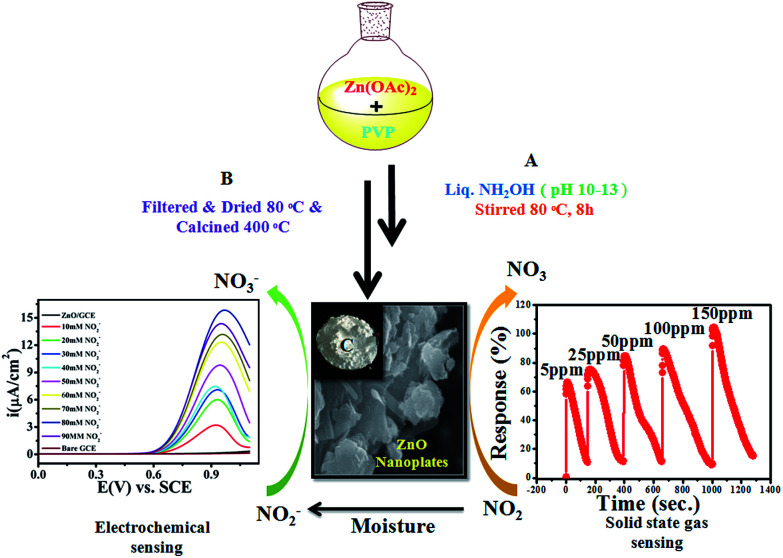
Synthesised ZnO NPs using a chemical approach for chemical and electrochemical NO_2_ sensing studies.

Historically, aqueous measurements of NO_2_ were taken, where in aqueous solution it is converted to nitrite (NO_2_^−^) ions.^19^ Unfortunately, nitrite (NO_2_^−^) is one of the well recognized and widespread inorganic pollutants present in industrial effluents and in environmental, food, industrial and physiological systems.^20–22^ It is toxic to humans and animals and, for example, NO_2_^−^ is converted to carcinogenic nitrosamines in the stomach.^23^ It irreversibly reacts with haemoglobin producing methemoglobin, a compound that reduces the oxygen transport capability of blood. Therefore, the development of new methods for the determination of NO_2_^−^ in food, water and biological fluids has received considerable attention by many researchers and these methods can give an indirect determination of NO_2_ gas.^24–28^ Various analytical methods have already been developed to determine NO_2_^−^ including colorimetry,^29^ chromatography,^30^ fluorometry,^31^ spectrophotometry^32^ and electrochemical methods^33–37^ for the quantitative and qualitative determination of NO_2_^−^. Among them, electrochemical methods have been well recognized because of their simple, easy to handle and controlled working. Therefore, as the aqueous form of NO_2_ is nitrite, our interest is focused on the determination of nitrite electrochemically in an aqueous system. Accordingly, in the present work, we proposed for first time a new and bifunctional ZnO based electrode system and direct chemical (electrical) and indirect (*via* nitrite) electrochemical (LSV) NO_2_ sensor studies with a low detection limit and high sensitivity are schematically shown in Scheme 1. Furthermore, the mechanistic pathway based on the combined results from chemical (electrical) and electrochemical (LSV) studies on a ZnO NP based bifunctional material towards electro-oxidation of NO_2_ and nitrite is also explored. The results obtained have demonstrated that the as-synthesised ZnO NP based bi-functional (chemical and electrochemical) sensor material exhibits high sensitivity, a low detection limit, a wide linear concentration range, and high stability and availability for accurate NO_2_ detection.

## 2 Experimental

### 2.1 Chemical

Zinc acetate 99.99% (Alfa-Aesar), PVP (polyvinylpyrrolidone), PVA (polyvinyl alcohol), sodium nitrite, pH 7.2 PBS, ammonia solution 25%, and acetone were procured from Sigma Aldrich. All other organic solvents were purchased at analytical grade and used without any further purification. Deionised water (18 MΩ) from a Milli-Q system was used for all syntheses and electrochemical evaluations of the electrocatalytic materials.

### 2.2 Synthesis of ZnO nanoplates (NPs)

ZnO NPs were synthesized by an inexpensive emulsion method using Zn(OAc)_2_·2H_2_O and poly-vinylpyrrolidone (PVP) as a source of Zn and as surface capping molecules, respectively. Briefly, the mixture of 100 mL deionised water with 0.1 M Zn (OAc)_2_·2H_2_O and a proportional amount of PVP was stirred vigorously at RT for 30 min. Furthermore, to this mixture ammonia solution was added drop-wise until the pH of the solution was 10–13, followed by heat treatment for the next 24 h at 80 °C which obtained a white milky precipitate. The obtained powder was washed several times with de-ionised water to remove water soluble side products and excess, unreacted ammonia until the complete removal obtained a neutral pH of the filtrate, followed by washing with methanol and finally the powder was dried at 100 °C for 1 h in a furnace. Finally, this as-synthesised powder was annealed at 400 °C for 1 h to remove solvent/organic impurities and was subsequently cooled to RT.

### 2.3 Material characterization

XRD patterns of the ZnO samples were recorded using an X-ray diffractometer with intense CuKα_1_ radiation (*λ* = 1.54 Å), at a scanning rate of 1 min^−1^ and in the scanning range of 2*θ* from 20–80°. The ZnO NPs were drop-coated over a carbon tape and the samples were then sputter-coated with Pt prior to their characterization using scanning electron microscopy (FE-SEM, JEOL, Japan) to avoid the charging effect. BET-analysis resulted in low pressure volumetric N_2_ adsorption–desorption measurements being performed at 77 K maintained by a low temperature liquid N_2_ bath, with pressure ranging from 0–760 torr using an AutosorbiQ (Quantachrome Inc., USA) gas sorption system. An out-gassing process was carried out at 200 °C for 15 h under dynamic vacuum (10^−3^ torr) until a constant weight was achieved. Ultrahigh purity grade (99.999%) N_2_ was used, which was further purified using calcium aluminosilicate adsorbents to remove trace amounts of water and other impurities prior to the measurements. For N_2_ isotherms, warm and cold free-space correction measurements were performed with ultrahigh pure He gas (99.999% purity). For the measurements, about 200 mg of the samples were used and to confirm complete removal of all guest H_2_O molecules from the samples, the weights of the samples were measured before and after out-gassing. The specific surface area of the ZnO NPs was calculated by the Brunauer–Emmett–Teller (BET) method. TGA thermograms were obtained using a TG Analyzer (SDT Q600 V20.9 Build 20) in the temperature range from 25 to 600 °C, with a heating rate of 10 °C min^−1^ in an air atmosphere.

### 2.4 NO_2_ gas sensing studies

Thin films of the ZnO NPs were tested for chemical solid state gas sensing. The electrical contacts of silver paste, separated by 1 cm, were coated onto the ZnO thin films. The sensor was mounted in a stainless steel test chamber (volume: 250 cm^3^). A desired concentration of the test gas in the chamber was achieved by injecting a known quantity of the gas using a micro-syringe. A change in resistance of the film as a function of time (response curve) was recorded at an operating temperature of 200 °C for 5, 25, 50, 100 and 150 ppm concentrations of NO_2_ gas, which was commercially procured. The response data was acquired using a computer interfaced Keithley-6514 electrometer. The recovery time of the sensor was recorded by exposing the sensor to fresh air. From the response curves, the response (*S*) was calculated using the equation:1
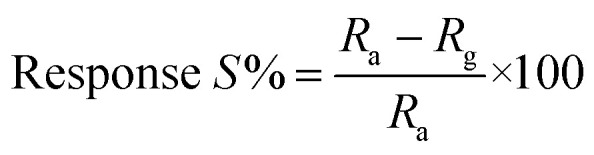
where *R*_g_ and *R*_a_ are resistances in the presence of the test gas (NO_2_) and in the absence of the test gas (in air), respectively.^38–41^ Response and recovery times were defined as the time needed for 90% of total resistance change upon exposure to gas and air, respectively.

### 2.5 Electrochemical studies

All electrochemical studies were performed on a CHI-660E (CH-instruments USA) instrument using a conventional three electrode test cell with a reversible calomel electrode (RCE) and platinum foil as the reference and counter electrodes, respectively. A 3 mm dia. glassy carbon (GC) electrode for the working electrode was polished using 0.3 and 0.05 μm alumina powders, followed by washing with water and methanol to remove inorganic and organic impurities. The working electrode was prepared as follows: a 10 μL aliquot of the slurry made by sonication of 1 mg of the ZnO NPs in 1 mL isopropyl alcohol was drop-casted onto the GC electrode. After this, 2 μL of 0.01 wt% Nafion, diluted with ethanol, was coated on the surface of the electrocatalytic ZnO NP layer to yield a uniform thin film. This electrode was then dried in air and was used as the working electrode for all electrochemical studies. An aqueous solution of 0.5 M phosphate buffer solution (PBS) was used as the electrolyte throughout the electrochemical studies. Furthermore, nitrite (NO_2_^−^) oxidation studies were performed using different concentrations of 10 to 90 mM NO_2_^−^ in 0.5 M PBS as the supporting electrolyte on ZnO NP based cost effective electrocatalytic systems. LSV of ZnO/GCE in 0.1 mol L^−1^ PBS (pH 7.2) containing 0.2 mol L^−1^ NO_2_^−^ was carried out at different scan rates (10–100 mV s^−1^). Furthermore, time dependent response curves for 10 mM nitrate in PBS (pH 7.2) at different time scales (30 s to 2 h 5 min) and at a scan rate of 50 mV s^−1^ were also obtained to determine the long term response of the ZnO NPs with 10 mM nitrate until all of the NO_2_^−^ species was used up. Moreover, prior to the electrochemical experiments, the electrolyte was dessicated with N_2_ gas. The electrocatalytic performance of these ZnO NPs was compared with GC (carbon form) instead of other metal systems.

## 3 Results and discussion

**Fig. 1:**
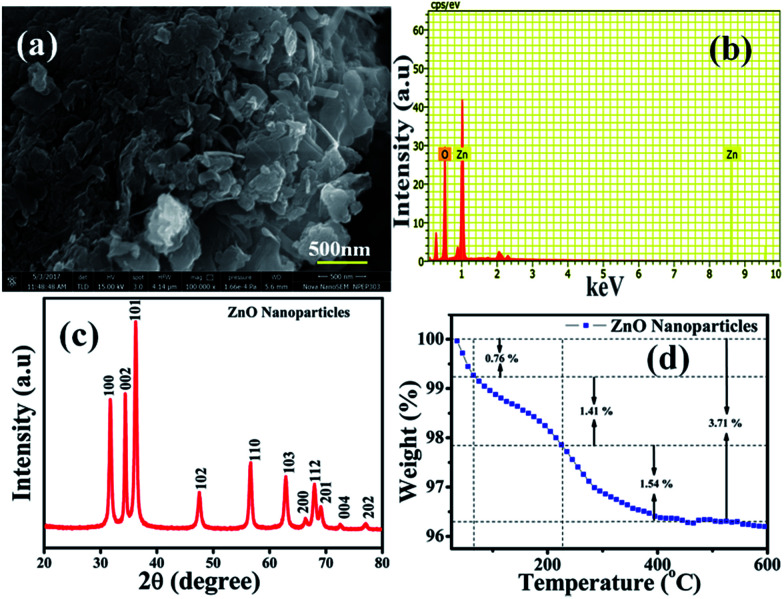
(a) Scanning electron microscopy (SEM) image of ZnO nanoplates (NPs) with edge width ∼100 nm. (b) EDAX of ZnO NPs confirms the presence of Zn and O only, (c) X-ray diffraction pattern (XRD) of (100), (002), (101), (102), (110), (103), (200), (112), (201), (004), and (202), corresponding to the ZnO NPs having a Wurtzite crystal structure, and (d) TGA of the ZnO NPs with three different losses corresponding to volatile solvent impurities along with moisture and surface bound PVP molecules, respectively.

The surface morphology of the chemically synthesised ZnO NPs was examined by SEM and is quite different from the results previously reported in the literature,^42^ which could be due to the role of the polyfunctional PVP molecules as surface directing molecules and the solution conditions used during nucleation resulting in growth in two dimensions (Fig. 1(a)). Eventually, large yields of homogenously distributed NPs were obtained. It should be noted that all the nanoplates are constructed individually and are distributed uniformly with similar and clear edges. The obtained dimension of the edge width of the ZnO NPs is ∼100 nm. Furthermore, the representative spot EDS analysis (Fig. 1(b)) shows signals at 1.02 keV and 8.76 keV corresponding to Zn and at 0.28 keV corresponding to O, clearly indicating that the nanoplates are formed of pure ZnO which is in good agreement with the literature.^43^ The XRD pattern shown in Fig. 1(c) illustrates the characteristic peaks of ZnO and represents its crystalline nature. Significantly, the peaks at 2*θ* values which correspond to the crystal planes of (100), (002), (101), (102), (110), (103), (200), (112), (201), (004), and (202) are in agreement with previous reports on a similar system^44^ and the diffraction peaks are in agreement with the JCPDS card no. 00-036-1451, which corresponds to the Wurtzite structure of ZnO. Moreover, no representative peaks for other impurity phases were detected, which confirms the high quality single phase ZnO NP formation. The average crystallite size (*d* = ∼100 nm) of the ZnO NPs was estimated by Scherrer’s formula and is in good agreement with morphological findings from SEM.

Moreover, Fig. 1(d) represents thermogravimetric analysis (TGA), which was performed to study the thermal stability of the as-synthesised ZnO NPs in an air atmosphere and shows weight loss in two steps. Significantly, the first step in the temperature range of 40–65 °C shows a weight loss of 0.76%, which could be attributed to the loss of volatile solvent molecules being trapped on/or within the surface adsorbed onto the ZnO in air dried samples.^45^ In the second step, 1.41% weight loss is observed between 65 to 226 °C and 1.54% weight loss is observed from 226 to 400 °C, corresponding to oxidative decomposition of surface bound PVP exponentially, which could be due to its poly-functional nature which helps to fix the temperature for further calcination processes. The calcination of ZnO in air at 400 °C results in a bright white powder, which indicates the complete decomposition of surface bound PVP, forming pure and PVP-free ZnO NPs. In summary, the TGA of the sample calcined in air shows that the mass ratio of ZnO NPs is 96.29% ZnO plus 3.71% PVP and water residues, and this temperature prolongs the features of the as-synthesised ZnO NPs.

**Fig. 2:**
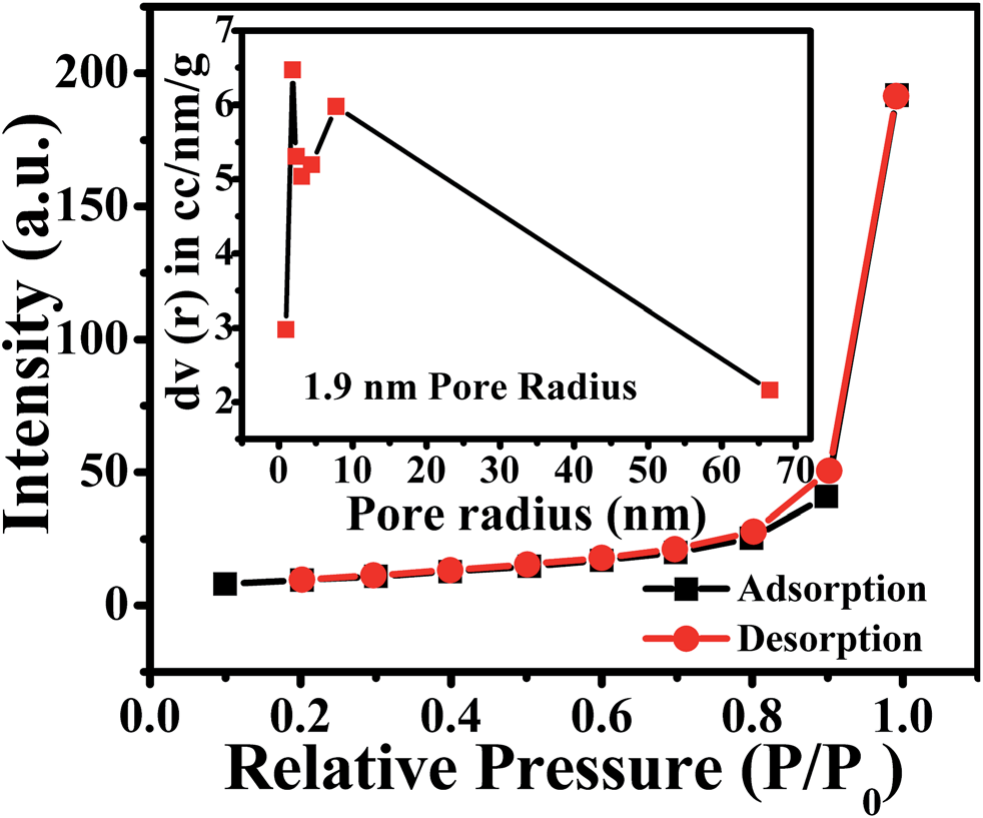
N_2_ adsorption (black)-desorption (red) isotherm and BJH pore size plot (inset) of annealed ZnO NPs.

Furthermore, a N_2_ adsorption-desorption isotherm of ZnO NPs was recorded to evaluate the available surface area for chemical and electrochemical interactive determination of NO_2_ and is shown in Fig. 2. The study reveals that ZnO displays a type II isotherm with a hysteresis loop at the relative pressure *P*/*P*_0_ ranging from 0.8 to 0.9. The characteristic loop indicates that the ZnO is a mesoporous material and the presence of mesopores can be attributed to the thermal decomposition of PVP (polyvinyl pyrrolidone) capped on the ZnO surface and the release of CO_2_ during calcinations.^46^ The BET (Brunauer–Emmett–Teller) surface area, calculated from the adsorption isotherm curve, is 27.77 m^2^ g^−1^. Moreover, the inset in Fig. 2 illustrates the corresponding pore size distribution plot calculated by BJH (Barrett–Joyner–Halenda) from the adsorption data. Significantly, the calculated average pore radius and pore volume of the samples were 1.9 nm and 6.4 × 10^−5^ cm^3^ g^−1^, respectively.

### 3.1 Solid state gas sensing

The gas-sensing performance of the chemically synthesised ZnO NPs was explored by injections of nitrogen dioxide (NO_2_) gas with different concentrations and also with different gases such as carbon monoxide (CO), sulfur dioxide (SO_2_), methanol (CH_3_OH), and chlorine (Cl_2_), simultaneously. The operational temperature was finalized on the basis of temperature dependent response studies for 100 ppm NO_2_ gas in the temperature range of 100–300 °C (shown in the ESI, Fig. S1†). Interestingly, the response is high for an operating temperature of 200 °C, hence this temperature was used for further studies. This high temperature response of ZnO NPs towards the chemical sensing of NO_2_ could be due to its semiconducting nature in a nanoregime.

**Fig. 3:**
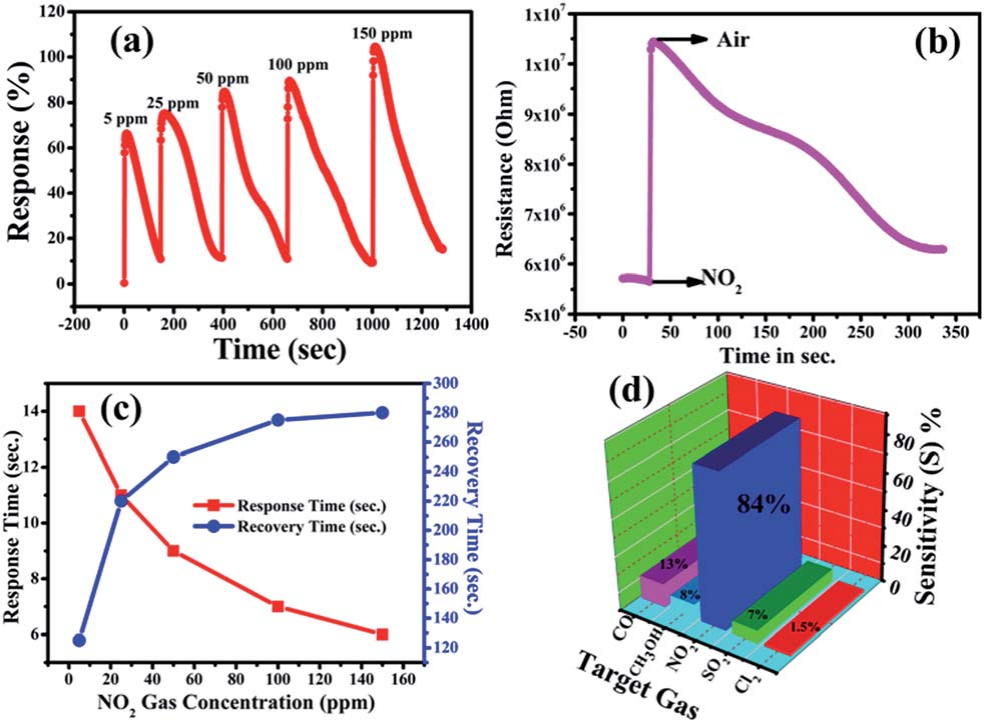
(a) Dynamic response of the ZnO NP based sensor at 5 ppm, 25 ppm and 50 ppm NO_2_ gas concentrations, simultaneously, (b) change in the resistance of the ZnO NP based film with respect to time upon the exposure of NO_2_ gas, (c) sensor response (red) and recovery (blue) times *versus* NO_2_ gas concentration and (d) gas selectivity of the ZnO sensor towards other gas species (CO, SO_2_, CH_3_OH, and Cl_2_).

Accordingly, Fig. 3(a) shows the response curve of the ZnO NP sensor with the various concentrations of NO_2_ gas at 200 °C, and it demonstrates that the response values of the sensor (calculated using eqn (1)) were found to increase with the increasing concentration of NO_2_ gas. Moreover, the response curve of the ZnO NP based sensor with 5, 25, 50, 100 and 150 ppm NO_2_ is shown in Fig. 3(a). The maximum response value of 105% at 150 ppm along with ∼66% at 50 ppm NO_2_ at 200 °C could be seen. At lower concentrations the NO_2_ gas molecules cover less of the surface of the ZnO NPs and, as a result, this lowers the surface interactions and consequently leads to a lower response. In addition, a high concentration of NO_2_ covers more and more of the surface of the ZnO NPs, thereby increasing the response value due to associated greater surface interactions.^47^

Fig. 3(c) shows the response and recovery times as functions of concentration of NO_2_ gas. The response time is shown to decrease with increasing gas concentration while the recovery time increases. This increase in time for recovery may be due to the heavier nature of NO_2_ and the reaction products, which delays the desorption of the species from the interface after the interaction, resulting in a decrease in desorption rate. The response time of the ZnO NP sensor was found to decrease from 14 s to 6 s, while the recovery time increased from 125 s to 280 s with the increase in concentration of NO_2_ from 5–150 ppm.^48^ The variations in time of response and recovery as a function of different NO_2_ concentrations (5–150 ppm) at 200 °C is shown in Fig. 3(c), which clearly indicates that the response and recovery times vary inversely with increasing concentrations of NO_2_ gas. Accordingly, the plot of the increase in the electrical resistance value of the ZnO NP based sensor upon the exposure of 150 ppm oxidizing NO_2_ gas at 200 °C is shown in Fig. 3(b).

Furthermore, Fig. 3(d) shows that an NO_2_ gas response of 84% is obtained at 50 ppm of different gas molecules. A selectivity study of the ZnO NPs was carried out with various target gases using a fixed 50 ppm concentration of each, and the observed responses are displayed in Fig. 3(d). The selectivity studies clearly suggest that the ZnO NPs are more sensitive towards NO_2_ gas in contrast to the other test gases: carbon monoxide (CO), sulfur dioxide (SO_2_), methanol (CH_3_OH), and chlorine (Cl_2_). The maximum selectivity towards NO_2_ might be due to a higher rate of interaction between the surface of the ZnO NPs and the NO_2_ gas molecules compared to all the other tested gases.^53^ It is known that the NO_2_ sensing mechanism of ZnO NPs depends on the active surface oxygen centres available on the surface of the ZnO NPs. Accordingly, Table 1 shows the comparison of the gas-sensing characteristics of the present work with those reported in the literature for NO_2_ gas in similar systems, and it concludes that our ZnO NPs have a comparatively better % gas response at lower gas concentrations and at a lower operating temperature.

**Table 1:** Comparison of performance of the as-synthesized ZnO NPs with some representative metal oxide based NO_2_ gas sensors from the literature

Materials (morphology)	Operating temperature (°C)	% gas response	Ref.
ZnO nanorod	225	35 at 50 ppm	49
ZnO nanoflowers	270	29 at 50 ppm	50
ZnO nanowire	225	50 at 50 ppm	51
ZnO nanostructures	200	9 at 20 ppm	52
ZnO NPs	200	66 at 5 ppm	Present work

### 3.2 Electrochemical detection of nitrite ions by ZnO electrodes

**Fig. 4:**
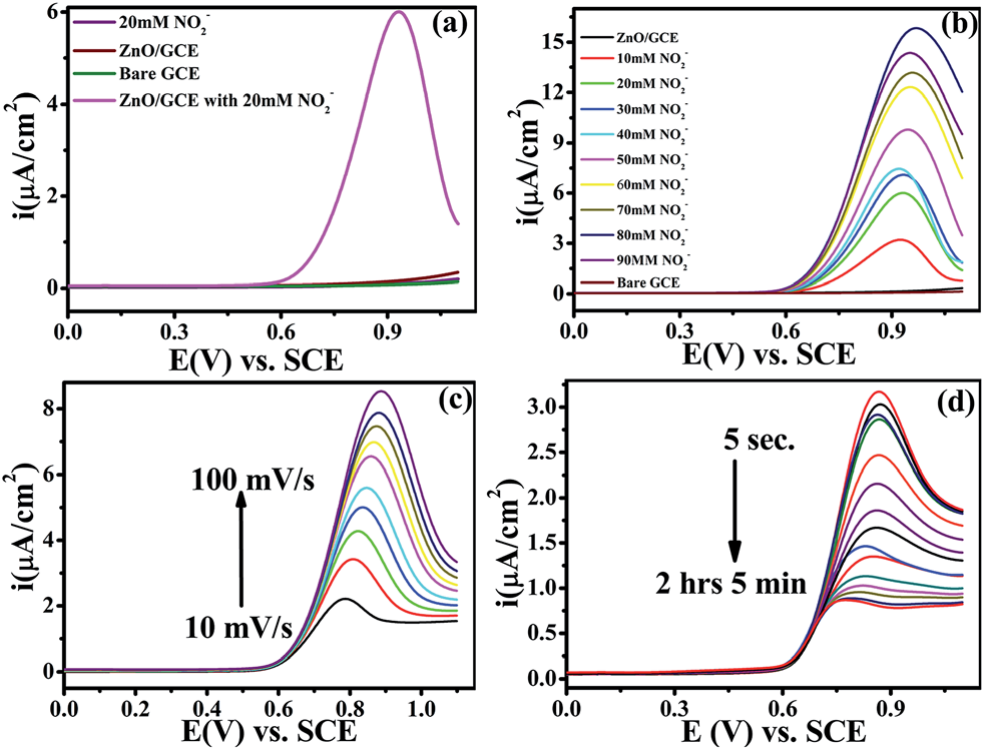
(a) Superimposed anodic segments for bare GC (green), GC modified by ZnO in phosphate buffer (red), and GC modified by ZnO with 20 mM nitrite in phosphate buffer (pH = 7.2) (pink). The GC blank with 20 mM nitrite in phosphate buffer (pH = 7.2) is shown in black. (b) LSV recorded for the ZnO/GCE electrode at various concentrations of NO_2_^−^ (10–90 mM) in PBS (pH 7.2) at a scan rate of 50 mV s^−1^. (c) LSV of ZnO/GCE in 0.1 mol L^−1^ PBS (pH 7.2) containing 0.2 mol L^−1^ NO_2_^−^ at different scan rates (10–100 mV s^−1^). (d) Time dependent response curves for 10 mM nitrate in PBS (pH 7.2) at different time scales (5 s to 2 h 5 min) at a scan rate of 50 mV s^−1^. For all electrochemical measurements Pt foil and calomel are the counter and reference electrodes, respectively.

Electrochemical studies were performed on a CHI-660E (CH-instruments USA) using a modified GC with ZnO NPs and Pt foil and SCE as counter and reference electrodes, respectively. Accordingly, Fig. 4(a) shows the superimposed LSV response for GC, GC-ZnO NPs in phosphate buffer (pH 7.2) solution, and GCE with 0.2 mM NO_2_^−^ in phosphate buffer and the LSV for GC-ZnO NPs with 0.2 mM NO_2_^−^ in phosphate buffer at a scan rate of 50 mV s^−1^ to correlate the potential range of surface ZnO-oxidation and NO_2_^−^ oxidation reactions. Accordingly, the superimposed LSV curves in phosphate buffer of the GC and GC-ZnO NPs electrodes, show that there are not any peaks in the absence of NO_2_^−^, while a new prominent oxidation peak obtained at +0.60 V *vs.* RCE in the presence of 20 mM NO_2_^−^, corresponding to the electrocatalytic oxidation of NO_2_^−^ to NO_3_^−^, is in line with similar reported systems. Moreover, no representative anodic peak is found in the anodic sweep on GC in the presence of NO_2_^−^ ions, which reflects its inability towards electro-oxidation of NO_2_^−^ (not shown for brevity). These findings significantly demonstrate how the electrocatalytic activity of ZnO NPs in an aqueous system may provide an indirect link *via* NO_2_^−^ oxidation and confirms the electrochemical ability of ZnO NPs towards NO_2_ determination. Furthermore, the influence of the increase in concentration of NO_2_^−^ on the electrocatalytic oxidation peak potential and peak current at the ZnO NP electrode in PBS (pH 7.2) was studied using an LSV curve (Fig. 4(b)). The oxidation peak current at 0.60 V *vs.* RCE shows a linear response with the concentration of NO_2_^−^ ions in the range of 10–90 mM and this linear range is broader than those of reported similar electrocatalytic systems.^54^ Furthermore, the influence of the scan rate on the electrocatalytic oxidation peak potential (*E*_pa_) and peak current for NO_2_^−^ at the ZnO/GCE electrode in 0.1 M PBS (pH 7.2) was studied using LSV, as shown in Fig. 4(c). The current values were found to increase with an increase in the scan rate from 10 to 100 mV s^−1^Fig. 4(c). The linear relationship between the anodic peak currents and the square root of the scan rate^55^ shows that electrooxidation of NO_2_^−^ is diffusion controlled. Furthermore, time dependent LSV with 10 mM nitrate in PBS (pH 7.2) takes almost 5 s to 2 h 5 min, confirmed by continuous LSV at a scan rate of 50 mV s^−1^, and the response curve is shown in Fig. 4(d). These findings support the bifunctional nature of ZnO NPs for the chemical determination of NO_2_ in solid state and the electrochemical determination of NO_2_^−^ in aqueous systems.

### 3.3 Presumable mechanism

The sensing mechanism involves the adsorption of oxygen species on the surface of the ZnO NPs and the abstraction of electrons, thus causing an increase in the potential barrier at the interfacial grain boundaries. The gas molecules interact with the oxygen species and produce a notable change in the electronic properties of the material. Therefore, the density of oxygen species on the surface defines the catalytic performance and hence the rate of reaction. More specifically, NO_2_ is an oxidizing gas with an electron affinity much higher than that of oxygen (0.48 eV). Thus, NO_2_ can interact with ZnO by trapping electrons directly through the surface oxygen ions, thereby generating new surface electron acceptor levels.^56,57^

The interaction of pre-adsorbed oxygen and NO_2_ molecules on the surface of ZnO is indicated in the equations:2
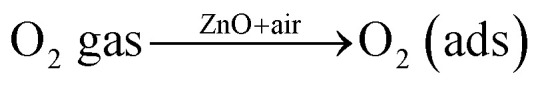
3
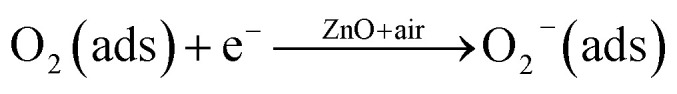
4
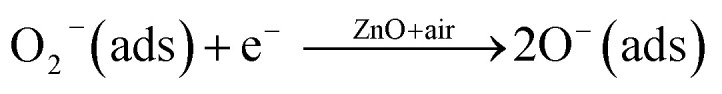

5 NO_2_ gas ↔ NO_2_(ads)

6 NO_2_(ads) + O_2_^−^(ads) + 2e^−^ ↔ NO_2_^−^(ads) + 2O^−^(ads)

These series of reactions result in the further decrease of electrons on the surface of the ZnO NPs, which leads to the increase in resistivity of the material. This change in resistivity can be used for the chemical detection and determination of NO_2_. There are many reports^58–64^ for enhanced gas sensing behaviours of ZnO based systems and the mechanism is explained by “spill-over effect”, which means that especially at the nano-regime ZnO NPs enhance the active surface with non-stoichiometric amounts of oxygen species. Moreover, the ZnO NPs in this work showed no evidence of forming metallic (Zn) particles either at the surface or in the interior of the ZnO NPs. This is confirmed by EDAX and TGA analysis and is in good agreement with previous reports.^65^ For example, the first principles calculation for the formation state of energies of ZnO reveals that it prefers the substitution site. Thus, in the present case, the enhancement in the response time is supposed to be explained by the structural defects generated in the ZnO lattice due to the nano-regime. Along with this, an acceptor-compensated charge transport mechanism is supposed to be responsible for the enhanced gas sensing behaviour.^66^ Selectivity is another important and significant sensor parameter to consider in the design of the sensor. In light of this, the response of the ZnO NP sensor towards a variety of gases including NO_2_, SO_2_, CO, Cl_2_ and CH_3_OH at 50 ppm and at a temperature of 200 °C was explored, to evaluate the sensor’s selectivity (Fig. 3(d)). Significantly, the gas sensor of ZnO NPs exhibits excellent selectivity towards NO_2_ gas in the presence of other reactive gases from the same family. These results suggest that the ZnO NP based sensor can be fabricated to monitor NO_2_ gas from polluted air. In summary, the NO_2_ sensing performance of the ZnO NP sensor was improved greatly; this study provides novel insights as well as aiding the development of next-generation chemical sensors for other gases.

**Scheme 2:**
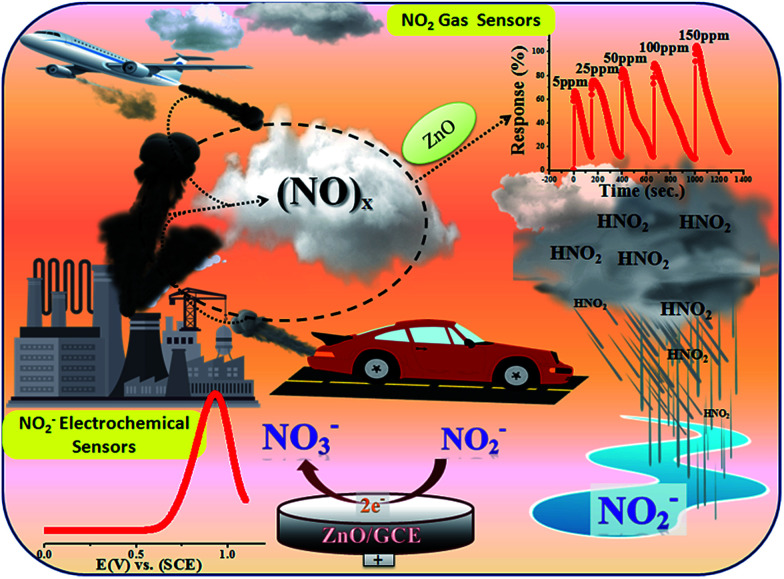
Schematic representation of the various steps employed for chemical (direct) and electrochemical (indirect) sensing of NO_2_.

In the atmosphere, NO_2_ and other oxides of nitrogen contribute to the formation of smog and acid rain, which results in water and soil pollution in addition to air pollution (schematically shown in Scheme 2). Accordingly, a great deal of this work was carried out to indirectly investigate the electrocatalytic oxidative transformation of NO_2_^−^ to NO_3_^−^ on ZnO NPs. In general, the uptake of NO_2_ into aqueous solution is considered to occur through a disproportionation reaction. In line with this, previous experimental results showed that NO_2_ to NO_2_^−^ conversion efficiency in an aqueous system is 100%.^67,68^

The electrocatalytic activity of the ZnO NPs with 20 mM of NO_2_^−^ in 0.1 M PBS (pH 7.2) was evaluated by cyclic voltammetry (CV). Fig. 4(a) shows the superimposed CV responses of different electrodes such as bare GCE, loaded ZnO and ZnO-GC in a 20 mM NO_2_^−^ solution. No signal was observed at the surface of GCE in the absence of nitrite. Interestingly, a prominent signal was observed at 0.84 V *vs.* SCE for the ZnO NPs with 20 mM NO_2_^−^ solutions. The ZnO NPs show a large anodic peak current corresponding to oxidation of NO_2_^−^. The corresponding mechanism of oxidation of NO_2_^−^ to NO_3_^−^ is as follows:HNO_2_ ⇋ NO_2_^−^ + H^+^NO_2_^−^ ⇋ NO_2_ + e^−^2NO_2_^−^ + H_2_O → NO_3_^−^ + NO_2_^−^ + 2H^+^

The enhancement in peak current indicates that the ZnO NP modified electrode could efficiently provide a large surface area and greater electrocatalytic oxidation of NO_2_^−^ Moreover, the ZnO NPs provide a high surface area with more adsorption sites for the adsorption of negatively charged ions of NO_2_^−^ due to its high species surface area (SSA). Therefore, a higher SSA leads to accumulation of more NO_2_^−^ ions on the surface of the electrodes resulting in the increase of electron transfer capacity.^69,70^ Accordingly, a comparison of the electrocatalytic performance of our ZnO NP based materials along with a list of some recently reported nanostructures is shown in Table 2.

**Table 2:** Performance comparison of NO_2_^−^ oxidation of the as-synthesised ZnO (our work) with other reported electrocatalytic systems from the literature

Sr. no.	Electrode	Detection limit (μM)	Ref.
1	f-ZnO@r-FGO	33	71
2	Gr-Nafion/GCE	11.61	72
3	Au-NPs/ethylenediamine/GCE	45	73
4	Nafion/SLGnP-TPA-Mb/GCE	10	74
5	Nano-Au/P3MT/GCE	2.3	75
6	ZnO/GCE	1	Present work

## 4 Conclusion

Herein, we have demonstrated the application of a cost effective colloidal chemical synthesis approach followed by heat treatment for the fabrication of ZnO NPs (edge thickness of ∼100 nm) which have a single phase Wurtzite crystal structure in a high yield. Thus the as-prepared ZnO NPs show a very efficient, facile, sensitive, selective and reproducible chemical and electrochemical sensing response for NO_2_ gas. The study suggests the usefulness of a ZnO sample because of the oxygen vacancies present. The ZnO sample shows a fast response time with a maximum gas response of around 105% at an NO_2_ gas concentration of 150 ppm, at 200 °C. Moreover, the ZnO NPs show a gas response of around 66% at very low concentrations (5 ppm) of NO_2_ gas. Furthermore, electrochemical studies were carried out for the indirect detection of NO_2_ by electrocatalytic oxidation of its representative aqueous NO_2_^−^ ions to NO_3_^−^ as a model reaction for fundamental investigations as well as for promising applications in electrochemical gas sensing technology. This ZnO NP based NO_2_^−^ sensor exhibits excellent analytical performance with long range potential, current and concentration stabilities and reproducible selective detection. Thus the as-synthesised ZnO NPs provide a bifunctional platform for chemical and electrochemical detection of NO_2_/NO_2_^−^. Moreover, further utilization of such a cost effective ZnO NP based nanosystem is also suitable for analysis of other toxic gases and biomolecules from biomass. Interestingly, these as-synthesised ZnO NPs are promising candidates for gas sensors, biosensors, electronics and catalysts in many more devices.

## Conflicts of interest

There are no conflicts to declare.

## Supplementary Material

RA-008-C8RA01358F-s001
