# Supplementary material for: Ultrasensitive and bifunctional ZnO nanoplates for an oxidative electrochemical and chemical sensor of NO2: implications towards environmental monitoring of the nitrite reaction
Source: RSC Adv. 2018 Mar 21;8(20):11177–85. doi: 10.1039/c8ra01358f (PMC9079129; doi:10.1039/c8ra01358f)
Supplement: RA-008-C8RA01358F-s001 [file RA-008-C8RA01358F-s001.pdf]

Electronic Supplementary Material (ESI) for RSC Advances.
This journal is © The Royal Society of Chemistry 2018

Supporting Information

Ultrasensitive and Bifunctional ZnO Nanoplates for Oxidative Electrochemical and Chemical Sensor of NO_2: Implications towards Environmental Monitoring of Nitrite Reaction

Shivsharan M. Mali,[a] Parag P. Chavan,[a] Yuvraj H. Navale,[b] Vikas B. Patil,[b] Bhaskar R. Sathe[a]*

[a]Department of Chemistry, Dr. Babasaheb Ambedkar Marathwada University, Aurangabad, 431004, Maharashtra, India

[b]Functional Materials Research Laboratory, School of Physical Sciences, Solapur University, Solapur, 413255, Maharashtra, India

Corresponding author E-mail: bhaskarsathe@gmail.com

SI1: Operational Temperature vs Response Studies:

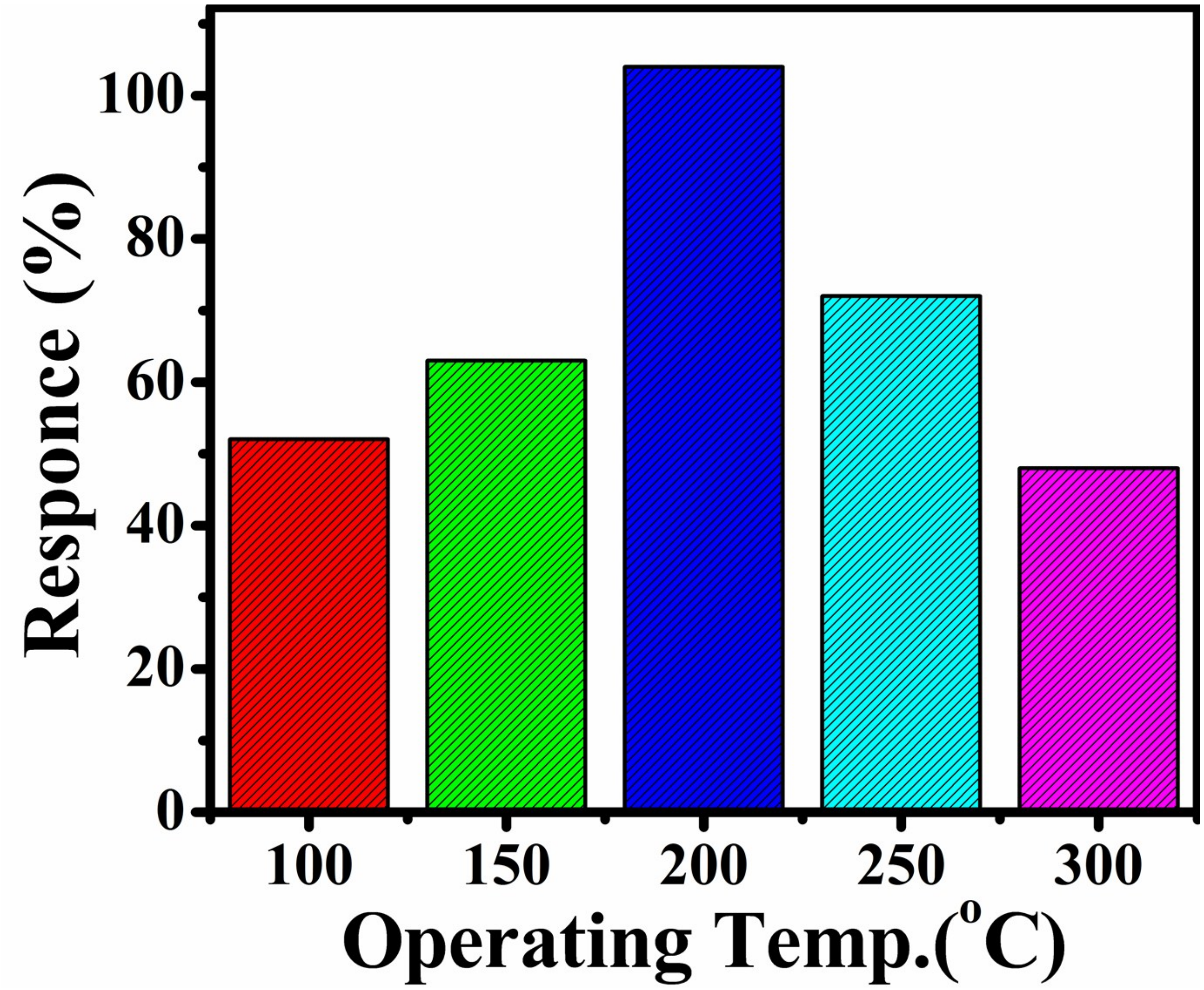

Fig S1: Operating temperature vs response (%) for 100 ppm NO_2 gas for different temperatures from 100- 300 °C.
